# Ensemble of Rotation Trees for Imbalanced Medical Datasets

**DOI:** 10.1155/2018/8902981

**Published:** 2018-04-10

**Authors:** Huaping Guo, Haiyan Liu, Chang-an Wu, Wei Liu, Wei She

**Affiliations:** ^1^School of Computer and Information Technology, Xinyang Normal University, Xinyang 464000, China; ^2^Cooper Innovation Center of Internet Healthcare, Zhengzhou University, Zhengzhou 450000, China; ^3^Department of Neurology, Xinyang Central Hospital, Xinyang 464000, China; ^4^School of Software Technology, Zhengzhou University, Zhengzhou 450001, China

## Abstract

Medical datasets are often predominately composed of “normal” examples with only a small percentage of “abnormal” ones and how to correctly recognize the abnormal examples is very meaningful. However, conventional classification learning methods try to pursue high accuracy by assuming that the number of any class examples is similar to each other, which lead to the fact that the abnormal class examples are usually ignored and misclassified to normal ones. In this paper, we propose a simple but effective ensemble method called ensemble of rotation trees (ERT) to handle this problem in imbalanced medical datasets. ERT learns an ensemble through the following four stages: (1) undersampling subsets from normal class, (2) obtaining new balanced training sets through combining each subset and abnormal class, (3) inducing a rotation matrix on randomly sampling subset of each new balanced set, and in each rotation matrix space, (4) learning a decision tree on each balanced training data. Here, the rotation matrix is mainly to improve the diversity between ensemble members, and undersampling technique aims to improve the performance of learned models on abnormal class. Experimental results show that, compared with other state-of-the-art methods, ERT shows significantly better performance for imbalanced medical datasets.

## 1. Introduction

In real world, the medical data often exists class imbalance, where the number of one class examples is larger than other classes [1, 2]. For two classes, the examples are usually categorized into normal (negative or majority) and abnormal (positive or minority) classes. The cost of misclassifying abnormal class examples is often higher than misclassifying the normal class ones. For instances, the “mammography” dataset contains 10,923 “healthy” patients and 260 “cancerous” patients and how to recognize the “cancerous” patients is very meaningful. However, traditional learning methods try to achieve high accuracy by assuming that the number of any class examples is similar to each other, which causes that the abnormal class examples are often overlooked and incorrectly classified as normal class [3, 4]. Therefore, many approaches have been proposed to tackle the problem.

Sampling technique including undersampling [5], oversampling [6], and SMOTE [7] is one of the most popular methods to solve the problem existing in imbalanced medical datasets. Undersampling technique is to learn models on the rebalanced dataset by sampling a subset of normal class and, unlike undersampling, oversampling rebalances the training dataset by repeating abnormal class examples [1]. SMOTE [7] is another version of oversampling technique, which generates new synthetic abnormal class examples by randomly interpolating pairs of closest neighbors of abnormal class.

Ensemble learning, which has often used to solute challenging issues when traditional classification models have been insufficient such as image detection [8–11], is another popular method to deal with imbalanced datasets. The proposed class imbalance-oriented ensemble learning methods can be mainly grouped into three categories: (1) bagging-, (2) boosting-, and (3) hybrid-based approaches. Both bagging- and boosting-based approaches often apply sampling technique to ensemble learning process, such as OverBagging, UnderBagging, UnderOverBagging [12], SMOTEBoost [13], and RUSBoost [14]. The former three methods combine bagging with sampling technique, and the latter two methods embed sampling technique into the process of learning each member. EasyEnsemble and BalanceCascade are the two specific examples of hybrid-based approaches [5]. EasyEnsemble undersamples several subsets from the normal class, trains a model using each of them, and combines the outputs of those models. The learning process of BalanceCascade is similar to EasyEnsemble with exception that in each step of training the models, the normal class examples, which are correctly classified by the current trained models, are removed from further consideration.

In this paper, we propose a novel ensemble method called ensemble of rotation trees (ERT) to build accurate and diverse classifiers to tackle class-imbalanced medical datasets. The main heuristics consist of (1) undersampling subsets from normal class, (2) obtaining new balanced training sets through combining each subset and abnormal class, (3) inducing a rotation matrix on randomly sampling subsets from each new balanced set, and in each rotation matrix space, (4) learning a decision tree on each balanced training data. Here, rotation matrix is to improve ensemble diversity, and undersampling technique mainly aims to improve the performance of learned models on abnormal class. The decision tree is selected as the chosen base model because it is sensitive to the rotation of feature axes, hence the name “rotation trees”. Compared with other state-of-the-art classification methods, ERT also shows a much better performance on class-imbalanced medical datasets.

This paper extends our previous work [15] in the following respects. First of all, it empirically compares a variety of ensemble method for medical datasets and this has led to new conclusions, such as the fact that the proposed ensemble significantly outperforms other ensemble methods for imbalanced medical datasets. The comparison is based on more medical datasets. Finally, this paper includes more discussion about why the proposed method works.

The rest of this paper is organized as follows: after presenting related work in Section 2, Section 3 describes the proposed learning method for medical datasets, Section 4 presents the experimental results, and finally, Section 5 concludes this work.

## 2. Strategies for Imbalanced Medical Datasets

In medical data analysis, it often happens that examples are categorized into an abnormal (minority or positive) group and a normal (majority or negative) group and the cost of misclassifying an abnormal example as a normal example is highly expensive. Take “mammography dataset” as an example. This dataset contains 10,923 “healthy” patients and 260 “cancerous” patients and a naive approach of classifying every example to a “healthy” patient would provide an accuracy of almost 97.68%. Although the naive approach achieves high accuracy, it incorrectly classifies all the “cancerous” patients.

Many techniques have been proposed to handle the imbalanced problem in medical datasets, where the efforts mainly focus on the methods of manipulating datasets and ensemble learning methods.

The methods of manipulating dataset are to rebalance the imbalanced medical data through manipulating data distribution such that traditional methods bias to abnormal class. Reported studies of manipulating datasets can be further subdivided two types: resampling and weighting the data space. Resampling techniques aim to alleviate the effect of class-imbalanced distribution through sampling data space to rebalance the corresponding imbalanced dataset. Commonly used sampling techniques are falling to the following three categories: oversampling methods, undersampling methods, and hybrid method. Oversampling techniques try to create new minority class examples to eliminate the harms of imbalanced problem. Randomly duplicating the minority samples and synthetic minority oversampling technique (SMOTE) [7] are the two most popular examples of oversampling techniques. Undersampling techniques, such as random undersampling (RUS) [5], the simplest yet most effective method, try to eliminate the harms of class-imbalanced distribution through removing the examples of the majority class. The hybrid method is a combination of oversampling and undersampling. The strategies of weighting data space adopt information concerning the misclassification costs to adjust the training set distribution, examples including cost-sensitive methods [16] and an ensemble of SVM with asymmetric misclassification costs [1].

Ensemble learning, which generally outperforms single classifiers in class-imbalanced problems [17], and decision trees are popular choices for the base classifiers in an ensemble [18]. According to Galar et al. [19], ensembles for class-imbalanced problem can be grouped into three categories: (1) bagging-, (2) boosting-, and (3) hybrid-based approaches. Bagging-based ensemble methods, such as UnderBagging, OverBagging, and UnderOverBagging [12], integrate bagging with resampling technique to improve model's performance on class-imbalanced problem, where UnderBagging uses undersampling technique to preprocess the training set before learning each member. On the contrary to UnderBagging, OverBagging uses oversampling technique instead of undersampling technique to preprocess the training set. UnderOverBagging uses both oversampling and undersampling techniques to adjust data distribution for training individual members. Boosting-based ensembles embed sampling techniques into the learning process of boosting algorithms: alter and bias the weight distribution to train the next classifier toward the abnormal class every iteration. For example, SMOTEBoost [13] uses SMOTE [7] to generate synthetic examples of abnormal class to alter data distribution, and RUSBoost [14] which performs similarly to SMOTEBoost uses RUS [5] to remove examples from the normal class to train base classifiers. Hybrid-based ensembles, such as EasyEnsemble and BalanceCascade [5], combine bagging with boosting (also with a sampling technique). Both EasyEnsemble and BalanceCascade use bagging as the main ensemble learning method and use AbaBoost as the base classifier learning method. The difference between these methods is the way in which they treat the normal class examples after each iteration. EasyEnsemble does not perform any operation after each AdaBoost iteration. Unlike EasyEnsemble, after learning an AdaBoost, BalanceCascade removes the normal class examples that are correctly classified with higher confidences from further consideration.

Rotation forest, an ensemble learning approach, often performances better than bagging and boosting due to build accurate and diverse classifiers by introducing subsets of features and rotation feature space [20]. This method is also applied to imbalanced problems, for example, Su et al. [21] employed class imbalance-oriented learner, namely, Hellinger distance decision tree (HDDT), as the base classifier of rotation forest to handle class-imbalanced problem, and each base classifier is constructed on the whole training set. Hosseinzadeh and Eftekharia [22] learned rotation forest on the data obtained by preprocessing training set using synthetic oversampling technique (SMOTE) and fuzzy cluster. Fang et al. [23] learned the rotation matrixes on datasets obtained by random undersampling or oversampling (SMOTE) the training set, and each base classifier is constructed on the whole training set.

This paper proposes a novel ensemble method for imbalanced medical datasets. Unlike bagging-, boosting-, and hybrid-based approaches, the proposed method learns each base classifier in rotation matrix space. Unlike conventional rotation forest-based approaches, the proposed method learns both rotation matrixes and base classifiers on the diverse balanced datasets instead of on imbalanced data or on the same data. More details are discussed in Section 3.

## 3. Ensemble of Rotation Trees for Imbalanced Medical Datasets

### 3.1. Ensemble of Rotation Trees

Class-imbalanced problem often exists in medical datasets. This problem causes that traditional classifier learning methods do not work well. This section proposes a novel ensemble method called ensemble of rotation trees (ERT) to handle imbalanced medical datasets. ERT learns an ensemble through the following two steps: (1) sampling subsets from normal class and learning a rotation matrix on each subset and (2) training a tree on the balanced dataset obtained from combining each subset and abnormal class set in the new feature space defined by current rotation matrix.

Let **x** = **[***x*_1_, *x*_2_,..., *x_n_*]*^T^* be an example of a medical dataset described by *n* features, and let ***X***_a_ be the abnormal class set in the form of *N*_a_ *× n* matrix and ***X***_n_ be the normal class set (in the form of *N*_n_ *× n* matrix). Denote by *h* ∈ *H* a classifier in the ensemble *H* and by *F*, the feature set. Like bagging, all classifiers can be trained in parallel. ERT constructs the current classifier *h* ∈ *H* using the following steps:

- *D = D*_n_ ∪ ***X***_a_, where *D*_n_ is a subset of ***X***_n_ obtained by randomly undersampling ***X***_n_ without replacement, and |*D*_n_| = |***X***_a_|.
- Split *F* randomly into subsets {***F****_j_*|*j* = 1, 2,…*n*/*L*}. The disjoint subsets are chosen to maximize the chance of high diversity.
- For each *F_j_*, draw a subset of size 50 percent from *D*. Run feature extraction method on *F_j_* and the subset to get feature projection components, **a**_*j*_^(1)^, **a**_*j*_^(2)^,…, **a**_*j*_^(*L*)^, each of size *L* × 1.
- Organize the components in a sparse “rotation” matrix ***R***

(1) $$\begin{array}{c} R=\left[\begin{array}{cccc} a_{1}^{\left(1\right)},a_{1}^{\left(2\right)},\ldots ,a_{1}^{\left(L\right)} & \left[0\right] & \cdots & \left[0\right]\\ \left[0\right] & a_{2}^{\left(1\right)},a_{2}^{\left(2\right)},\ldots ,a_{2}^{\left(L\right)} & \cdots & \left[0\right]\\ \vdots & \vdots & \ddots & \vdots \\ \left[0\right] & \left[0\right] & \cdots & a_{n/L}^{\left(1\right)},a_{n/L}^{\left(2\right)},\ldots ,a_{n/L}^{\left(L\right)} \end{array}\right] \end{array}$$

(v) Train current classifier *h* using *D**R**.*

Pseudocode 1 shows the pseudocode for the algorithm of ERT. The differences with rotation forest-based class-imbalanced methods (refers to Section 2) are mainly reflected in lines 4~5 and lines 14~15. Lines 4~5 construct new balanced training set *D_i_* through undersampling subset *D*_n_ from the normal set ***X***_n_ with the size of equal to that of abnormal set ***X***_a_. Lines 14~15 learn base classifier *h_i_* on the balanced data *D_i_* (obtained in steps 4~5) in matrix space ***R****_i_* through projecting *D_i_* using ***R****_i_* to obtain a new balanced training set *D*_*i*, train_ *= D_i_****R****_i_*. Therefore, both the rotation matrix ***R****_i_* and base classifier *h_i_* are learned from balanced dataset. Besides, unlike conventional rotation forest-based methods, which select and eliminate a random nonempty subspace of classes, ERT does not handle classes due to only two classes used in this paper.

In this paper, we chose decision trees as the base classifiers because they are sensitive to the rotation of the feature axes and still can be very accurate. The feature extraction is based on principal component analysis (PCA) [24] following rotation forest [20]. The running time of ERT is mainly dominated by constructing decision trees, running PCA, and rotating the datasets. Therefore, the computational complexity of ERT is the same to rotation forest [13].

### 3.2. Discussion

Two issues in ensemble should be addressed for imbalanced medical datasets: high performance of individual ensemble member bias towards abnormal class and the diversity between the members. Undersampling technique is employed to normal class such that individual base classifiers focus more on abnormal class. Specifically, ERT (the proposed method) undersample normal class set such that the learned rotate matrixes capture more on the distribution of the abnormal class set, which enhances the performance of individual classifiers on abnormal class (line 4, Pseudocode 1). Besides, ERT learns each individual classifier on rebalanced dataset obtained by undersampling the training set (lines 15, Pseudocode 1).

Diversity is one major issue to the success of an ensemble, and the intended diversity in the proposed model comes from the following two approaches: (1) the undersampling technique used to sample the normal class (refer to line 4 in Pseudocode 1) and (2) the difference in the possible feature subsets (refer to lines 6–14 in Pseudocode 1). For the first method, the larger the ratio between the size of normal class set and abnormal class set, the larger the diversity of individual classifiers. For the second approach, the number of different partitions of the feature set into *n*/*L* subsets is

(2) $$\begin{array}{c} T=\frac{n!}{\left(K\right)!{\left(L!\right)}^{n/L}}. \end{array}$$

For the ensemble with *M* members, the probability of all the members be different can be calculated by

(3) $$\begin{array}{c} P\left(different\;classifiers\right)=\frac{T!}{\left(T-M\right)!T^{L}}. \end{array}$$

For example, the probability that all different classifiers of an ensemble with 50 member for *n* = 9 is less than 0.01, and thus, an extra randomization of the ensemble is meaningful, especially for balanced datasets. Following rotation forest [20], we draw a bootstrap sample of objects, and PCA was applied on the subset.

## 4. Experiments

### 4.1. Evaluation Metrics

Evaluation metric is extremely essential to assess the effectiveness of an algorithm, and traditionally, accuracy is the most frequently used one. The examples classified by a classifier can be grouped into four categories as shown in Table 1, and thus, accuracy is defined as

(4) $$\begin{array}{c} Accuracy=\frac{TA+TN}{TA+TN+FA+FN}. \end{array}$$

However, accuracy is inadequate for imbalanced medical problem and other metrics are proposed, including precision, recall, f-measure, g-mean, and AUC. Precision and recall are, respectively, designed as

(5) $$\begin{array}{c} Precision=\frac{TA}{TA+FA},\\ Recall=\frac{TA}{TA+FN}. \end{array}$$

F-measure is a harmonic mean between recall and precision. Specifically, f-measure is defined as

(6) $$\begin{array}{c} f‐measure=\frac{\left(1+\delta^{2}\right)\times recall\times precision}{\delta^{2}\times recall+precision}, \end{array}$$

where *δ*, often set to be 1, is a coefficient to adjust the relative importance of precision versus recall.

Like f-measure, g-mean is another metric considering both normal class and abnormal class. Specifically, g-mean measures the balanced performance of a classifier using the geometric mean of the recall of abnormal class and that of normal class. Formally, g-mean is as follows:

(7) $$\begin{array}{c} g‐mean=\sqrt{\frac{TA}{TA+FN}\times \frac{TN}{TN+FA}}. \end{array}$$

Besides, AUC is a commonly used measure to evaluate models' performances. According to [25], AUC can be estimated by

(8) $$\begin{array}{c} AUC=\frac{\left(\left(TP/TP+FN\right)+\left(TN/TN+FP\right)\right)}{2}. \end{array}$$

In this paper, we employ recall, f-measure, g-mean, and AUC to evaluate the classification performance on imbalanced datasets.

### 4.2. Datasets and Experimental Setup

Eight medical datasets are selected in this paper. All the datasets are two-class imbalanced medical datasets [26]. The imbalanced degree of these dataset varies from 0.061 (highly imbalanced) to 0.349 (only slightly imbalanced), where imbalanced degree is defined as the ratio of the size of the abnormal class to that of the normal class. The details of the datasets are shown in Table 2, where #Degree is the imbalance degree, #Size is the size of datasets, and #Attrs is the number of attributes.

A 10-fold cross-validation [27] is performed to test model performance: each dataset is randomly divided into tenfolds. For each fold, the other ninefolds are used to train a model, and the current fold is to test the model. We run ten times of the 10-fold cross-validation, and therefore, 100 models are constructed for each dataset.

To evaluate the performance of ERT (the proposed method), we compare it with RURF [23], EasyEnsemble [5], BalanceCascade [5], Bagging [28], and C4.5 [29]:

- RURF is a class imbalance-oriented version of rotation forest (RF) which learns projection matrixes on random undersampling (RU) datasets. C4.5 was selected as the base learner and the number of the base classifiers was set to be 100.
- EasyEnsemble samples *T* subsets from the normal class and uses AdaBoost with C4.5 as the weak learner to learn *M* base classifiers on each subset. We set *T* = *M* = 10 and therefore 100 trees are learned.
- BalanceCascade is similar to EasyEnsemble except that it removes major class examples that are correctly classified by trained learners from further consideration. *T* and *M* are both set to be 10 and therefore 100 trees are learned.
- Bagging learns each base classifier on a resampled dataset. C4.5 is set to be the weak classifier and the number of base classifiers is set to be 100.
- ERT is the proposed method in this paper. Here, we set *M* = 100, namely, the number of bases classifier is 100. C4.5 is used to train base classifiers (refer to Pseudocode 1).

### 4.3. Experimental Results

**Figure 1:**
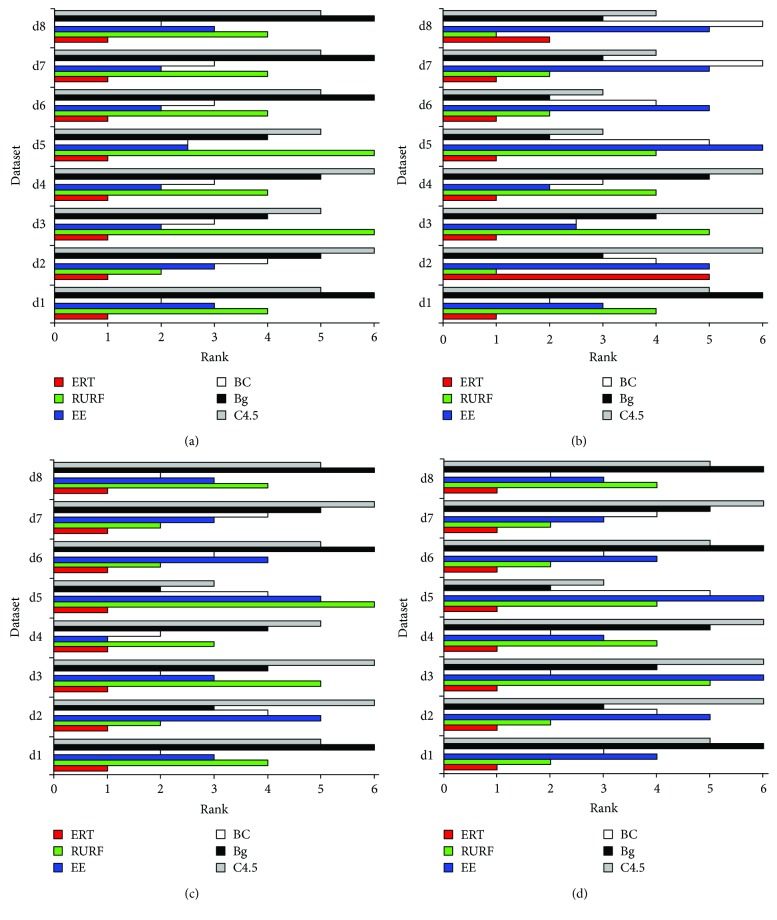
The ranks of methods on measures of (a) recall, (b) f-measure, (c) g-mean, and (d) AUC, where ERT, EE, BC, and Bg indicate ERT, EasyEnsemble, BalanceCascade, and Bagging, respectively.

To evaluate the performance of ERT (the proposed method), ERT is compared with RURF, EasyEnsemble, BalanceCascade, Bagging, and C4.5 (more details refer to Section 4.2). The corresponding results are reported both in tables and one figure, where four tables report the results of the eight comparing methods on the measures of recall, f-measure, g-mean, and AUC, and the figure reports the ranks of the methods on recall, f-measure, g-mean, and AUC. In these tables, a bullet (an open circle) next to a result indicates that ERT significantly outperforms (is outperformed by) the respective method (column) for respective dataset (row) in pairwise *t*-test at 0.05 significance level. The last rows in these tables are the average results. The ranks of these methods on measure of recall, f-measure, g-mean, and AUC shown in Figure 1 are calculated as follow [30, 31]: on a dataset, the best performing algorithm gets the rank of 1.0, the second best gets the rank of 2.0, and so on. In case of ties, average ranks are assigned.

Table 3 and Figure 1(a) show the summarizing results and the ranks of the six comparing methods on measure of recall, respectively. From Table 3, ERT significantly outperforms both bagging and C4.5 on all the eight medical datasets, and the average recall of ERT is 0.2087 higher than C4.5 (recall ∈ [0, 1]). Also, ERT statistically outperforms RURF, EasyEnsemble, and BalancedCascade on eight, seven, and six out of the datasets, respectively, and outperforms them on all datasets. Besides, from Figure 1(a), we observe that the average ranks of ERT, RURF, EasyEnsemble, BalanceCascade, bagging, and C4.5 are 1.0, 4.3, 2.4, 2.8, 5.3, and 5.3, respectively.

Table 4 and Figure 1(b) illustrate the summarizing results and the ranks of ERT, RURF, EasyEnsemble, BalanceCascade, Bagging, and C4.5 on f-measure, respectively. From Table 4, ERT shows much better performance comparing to other methods. Specifically, ERT statistically outperforms RURF, EasyEnsemble, and BalanceCascade on four, eight, eight, seven, and seven out of the eight datasets. Figure 1(b) shows that ERT wins on six, eight, eight, seven, and seven out of the eight datasets. Besides, ERT is statistically outperformed by RURF, bagging, and C4.5 on “sick.” Combining the results of Table 3 and Figure 1(a), we have that ERT obtains high recall by scarifying the precision of models on “sick.”

G-mean summaries and the corresponding ranks of ERT, RURF, EasyEnsemble, BalanceCascade, Bagging, and C4.5 are reported in Table 5 and Figure 1(c), respectively. Table 5 shows that ERT significantly outperforms RURF, EasyEnsemble, BalanceCascade, Bagging, and C4.5 on all of the eight datasets, and Figure 1(c) shows that ERT ranks first with average rank 1.0, followed by BalanceCascade (2.9), EasyEnesemble (3.4), RURF(3.5), Bagging (4.5), and C4.5 (5.13).

Table 6 and Figure 1(d) depict AUC and the ranks of ERT, RURF, EasyEnsemble, BalanceCascade, Bagging, and C4.5, respectively. Similar to the results on g-mean, ERT significantly wins on all the eight sets comparing to other methods. The average AUC (ranks) of ERT, RURF, EasyEnsemble, BalanceCascade, Bagging, and C4.5 are 0.8573(1.0), 0.8093(3.1), 0.8096(4.3), 0.8098(3.1), 0.7959(4.6), and 0.7899(3.3), respectively.

## 5. Conclusion

In this paper, we propose a novel method called ensemble of rotation trees (ERT), which aims to build accurate and diverse classifiers to handle imbalanced medical data. The main heuristic consists of (1) sampling subsets from normal class, (2) learning a rotation matrix on each subset, and (3) learning a tree using each subset and abnormal class set in the new feature space. Experimental results show that ERT performs better than other state-of-the-art classification methods on measure of recall, f-measure, g-mean, and AUC on medical datasets.

## Figures and Tables

**Pseudocode 1:**
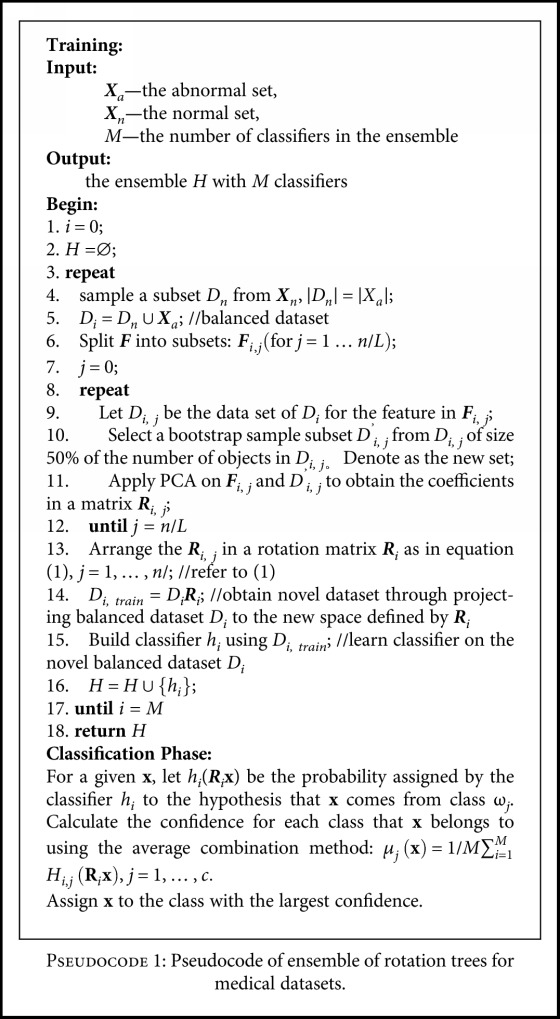
Pseudocode of ensemble of rotation trees for medical datasets.

**Table 1:** Confusion matrix.

	Predicted as abnormal	Predicted as normal
Actually abnormal	TA	FN
Actually normal	FA	TN

**Table 2:** The dataset used in this paper.

ID	Datasets	#Degree	#Size	#Attrs
d1	Breast-cancer	0.297	286	10
d2	Breast-wisconsin	0.345	699	11
d3	Diabetes	0.349	768	9
d4	Hepatitis	0.206	155	20
d5	Lymphography-normal-fibrosis	0.0405	148	19
d6	New-thyroid1	0.162	215	6
d7	New-thyroid2	0.162	215	6
d8	Sick	0.061	3772	30

**Table 3:** The recalls and standard errors of ERT, RURO, EasyEnsemble, BalanceCascade, Bagging, and C4.5.

Dataset	ERT	RURF	EasyEnsemble	BalanceCascade	Bagging	C4.5
d1	0.5917 ± 0.1733	0.2975 ± 0.1572●	0.4476 ± 0.1687●	0.4522 ± 0.1607●	0.2433 ± 0.1362●	0.2471 ± 0.1441●
d2	0.9946 ± 0.0140	0.9847 ± 0.0261●	0.9643 ± 0.0382●	0.9660 ± 0.0379●	0.9502 ± 0.0487●	0.9198 ± 0.0492●
d3	0.7834 ± 0.0681	0.5528 ± 0.0846●	0.7828 ± 0.0766●	0.7824 ± 0.0777●	0.6110 ± 0.0912●	0.5915 ± 0.1178●
d4	0.8150 ± 0.2265	0.4792 ± 0.2728●	0.7742 ± 0.2285●	0.7658 ± 0.2302●	0.3642 ± 0.2757●	0.3442 ± 0.2618●
d5	0.5300 ± 0.5016	0.2300 ± 0.4230●	0.3000 ± 0.4606●	0.3000 ± 0.4606●	0.2900 ± 0.4560●	0.2800 ± 0.4513●
d6	0.9975 ± 0.0250	0.9242 ± 0.1382●	0.9350 ± 0.1311●	0.9342 ± 0.1373●	0.8692 ± 0.1758●	0.8983 ± 0.1767●
d7	0.9975 ± 0.0250	0.9158 ± 0.1404●	0.9467 ± 0.1217●	0.9433 ± 0.1247●	0.8750 ± 0.1893●	0.8775 ± 0.1912●
d8	0.9861 ± 0.0230	0.8884 ± 0.0651●	0.9805 ± 0.0303●	0.9814 ± 0.0289	0.8658 ± 0.0760●	0.8684 ± 0.0717●
Average	0.8370	0.6591	0.7664	0.7657	0.6336	0.6283

●: ERT is significantly better; level of significance: 0.05.

**Table 4:** The f-measures and standard errors of ERT, RURO, EasyEnsemble, BalanceCascade, Bagging, and C4.5.

Dataset	ERT	RURF	EasyEnsemble	BalanceCascade	Bagging	C4.5
d1	0.5031 ± 0.1159	0.3893 ± 0.1756●	0.4407 ± 0.1314●	0.4453 ± 0.1225●	0.3383 ± 0.1672●	0.3415 ± 0.1702●
d2	0.9587 ± 0.0234	0.9607 ± 0.0250	0.9328 ± 0.0311●	0.9350 ± 0.0312●	0.9429 ± 0.0346●	0.9171 ± 0.0369●
d3	0.6884 ± 0.0528	0.6196 ± 0.0695●	0.6749 ± 0.0518●	0.6749 ± 0.0519●	0.6434 ± 0.0722●	0.6148 ± 0.0836●
d4	0.6227 ± 0.1668	0.5117 ± 0.2505●	0.5673 ± 0.1808●	0.5632 ± 0.1847●	0.4154 ± 0.2792●	0.3856 ± 0.2671●
d5	0.3497 ± 0.3659	0.2300 ± 0.4230●	0.1019 ± 0.1694●	0.1043 ± 0.1775●	0.2900 ± 0.4560●	0.2800 ± 0.4513●
d6	0.9483 ± 0.0731	0.9408 ± 0.0972	0.8831 ± 0.1293●	0.8881 ± 0.1345●	0.8987 ± 0.1299●	0.8974 ± 0.1356●
d7	0.9506 ± 0.0669	0.9410 ± 0.0910	0.8623 ± 0.1332●	0.8572 ± 0.1329●	0.8912 ± 0.1387●	0.8762 ± 0.1424●
d8	0.8046 ± 0.0492	0.9168 ± 0.0439○	0.7682 ± 0.0557●	0.7674 ± 0.0562●	0.8991 ± 0.0516○	0.8878 ± 0.0532○
Average	0.7283	0.6887	0.6539	0.6544	0.6649	0.6501

●: ERT is significantly better; ○: ERT is significantly worse; level of significance: 0.05.

**Table 5:** The g-means and standard errors of ERT, RURO, EasyEnsemble, BalanceCascade, Bagging, and C4.5.

Dataset	ERT	RURF	EasyEnsemble	BalanceCascade	Bagging	C4.5
d1	0.6289 ± 0.0991	0.4993 ± 0.1640●	0.5726 ± 0.1133●	0.5767 ± 0.1046●	0.4470 ± 0.1722●	0.4507 ± 0.1713●
d2	0.9756 ± 0.0144	0.9747 ± 0.0174	0.9544 ± 0.0229●	0.9560 ± 0.0231●	0.9576 ± 0.0280●	0.9366 ± 0.0291●
d3	0.7573 ± 0.0463	0.6945 ± 0.0548●	0.7443 ± 0.0461●	0.7444 ± 0.0460●	0.7173 ± 0.0575●	0.6947 ± 0.0685●
d4	0.7848 ± 0.1580	0.6122 ± 0.2577●	0.7451 ± 0.1567●	0.7391 ± 0.1684●	0.4999 ± 0.3021●	0.4759 ± 0.2990●
d5	0.5036 ± 0.4774	0.2300 ± 0.4230●	0.2440 ± 0.3786●	0.2445 ± 0.3792●	0.2900 ± 0.4560●	0.2800 ± 0.4513●
d6	0.9873 ± 0.0206	0.9553 ± 0.0777●	0.9449 ± 0.0784●	0.9457 ± 0.0832●	0.9224 ± 0.1040●	0.9334 ± 0.1055●
d7	0.9879 ± 0.0193	0.9520 ± 0.0779●	0.9431 ± 0.0725●	0.9408 ± 0.0734●	0.9226 ± 0.1125●	0.9203 ± 0.1131●
d8	0.9775 ± 0.0128	0.9404 ± 0.0352●	0.9710 ± 0.0159●	0.9713 ± 0.0151●	0.9279 ± 0.0414●	0.9284 ± 0.0394●
Average	0.8254	0.7323	0.7649	0.7648	0.7106	0.7025

●: ERT is significantly better; level of significance: 0.05.

**Table 6:** The AUCs and standard errors of ERT, RURO, EasyEnsemble, BalanceCascade, Bagging, and C4.5.

Dataset	ERT	RURF	EasyEnsemble	BalanceCascade	Bagging	C4.5
d1	0.6404 ± 0.0906	0.6117 ± 0.0846●	0.6078 ± 0.0872●	0.6099 ± 0.0828●	0.5929 ± 0.0719●	0.5944 ± 0.0755●
d2	0.9759 ± 0.0141	0.9750 ± 0.0171	0.9548 ± 0.0226●	0.9564 ± 0.0228●	0.9580 ± 0.0275●	0.9372 ± 0.0287●
d3	0.7592 ± 0.0458	0.7157 ± 0.0459●	0.7472 ± 0.0462●	0.7473 ± 0.0460●	0.7295 ± 0.0511●	0.7099 ± 0.0569●
d4	0.8039 ± 0.1158	0.6992 ± 0.1415●	0.7590 ± 0.1372●	0.7559 ± 0.1364●	0.6502 ± 0.1416●	0.6376 ± 0.1272●
d5	0.7258 ± 0.2437	0.6150 ± 0.2115●	0.5429 ± 0.2065●	0.5445 ± 0.2060●	0.6446 ± 0.2283●	0.6396 ± 0.2259●
d6	0.9876 ± 0.0198	0.9590 ± 0.0701●	0.9478 ± 0.0735●	0.9488 ± 0.0774●	0.9299 ± 0.0900●	0.9403 ± 0.0896●
d7	0.9882 ± 0.0186	0.9560 ± 0.0705●	0.9464 ± 0.0656●	0.9442 ± 0.0666●	0.9311 ± 0.0954●	0.9285 ± 0.0972●
d8	0.9776 ± 0.0128	0.9426 ± 0.0326●	0.9712 ± 0.0158●	0.9715 ± 0.0151●	0.9310 ± 0.0382●	0.9314 ± 0.0360○
Average	0.8573	0.8093	0.8096	0.8098	0.7959	0.7899

●: ERT is significantly better; ○: ERT is significantly worse; level of significance: 0.05.
